# The effects of spaced transcranial Direct Current Stimulation combined with conventional dysphagia therapy in Parkinson’s disease: A case report

**DOI:** 10.17179/excli2020-2434

**Published:** 2020-06-04

**Authors:** Ali Akbar Dashtelei, Michael A. Nitsche, Jalal Bakhtiari, Seyed Amirhassan Habibi, Mojtaba Sepandi, Ahmad Reza Khatoonabadi

**Affiliations:** 1 Speech Therapy Department, Tehran University of Medical Sciences, Tehran, Iran; 2 Department Psychology and Neurosciences, Leibniz Research Centre for Working Environment and Human Factors, Dortmund, Germany; 3 Department of Neurology, University Medical Hospital Bergmannsheil, Bochum, Germany; 4 Speech Therapy Department, Semnan University of Medical Sciences, Tehran, Iran; 5 Department of Neurology, Rasoul Akram Hospital, Iran University of Medical Science, Tehran, Iran; 6 Department of Epidemiology and Biostatistics, Baqyiatallah University of Medical Science, Tehran, Iran; 7 Health Research Center, Life Style Institute, Baqyiatallah University of Medical Science, Tehran, Iran

**Keywords:** Parkinson's disease, dysphagia, transcranial Direct Current Stimulation, non-invasive brain stimulation, swallowing rehabilitation

## Abstract

Parkinson's disease (PD) is a neurodegenerative disease of the central nervous system that is characterized by a set of motor and non-motor symptoms. Impaired swallowing or dysphagia is one relatively common motor symptom in patients with PD. We investigated whether neuroplasticity induction by spaced transcranial Direct Current Stimulation (tDCS) combined with conventional swallowing therapy leads to long-lasting effects on swallowing ability in patients with PD. We present a case of a 61-year-old male PD patient with dysphagia. Conventional Swallowing Therapy (CDT) combined with tDCS (bilateral anodal, 1 mA, 20 min, 10 online sessions, twice daily with a 20 min interval in between for five days over two weeks) was applied over the pharyngeal motor cortex. Our findings suggest that anodal tDCS combined with CDT is feasible, safe, and well-tolerated, and leads to a clinically relevant improvement of swallowing functions.

## Introduction

Impaired swallowing or dysphagia is one of the most common motor symptoms in patients with Parkinson's disease (PD). Its prevalence varies within 18.5 % to 100 % in different studies. One of the main medical complications is aspiration pneumonia, which can be life-threatening (Baijens and Speyer, 2009[1]; Tarameshlu et al., 2019[10]).

Recently, non-invasive brain stimulation techniques, including transcranial direct current stimulation (tDCS), were introduced in swallowing rehabilitation treatment. The rationale for application of this technique is an enhancement of rehabilitation task-related long term potentiation with the aim to improve cortical re-organization of swallowing functions. Anodal tDCS-induced neuroplasticity is driven by glutamatergic and calcium-dependent processes and enhances motor task-related activity of brain networks. Multiple-session interventions can lead to long-lasting behavioral effects (Triccas et al., 2016[11]). As an innovative therapeutic approach in PD, we hypothesized that combining anodal tDCS with swallowing therapy over multiple sessions to induce long-lasting effects (e.g. late phase plasticity), which are clinically relevant, would have a prolonged beneficial effect on dysphagia in PD.

## Case Presentation

The patient was a 61-year-old male with idiopathic Parkinson's disease diagnosed nine years ago. His medical history showed only hypertension and no other diseases except PD. The patient was managing his daily routine independently. PD was treated pharmacologically by 100 mg L-DOPA (125 mg Madopar), three times a day for the last 18 months. His baseline Mini-Mental Status Exam (MMSE) score was 25. The motor examination showed mild bradykinesia of all limbs and resting tremor of the left hand. The patient was in stage 2, according to the Modified Hoehn and Yahr Disability Scale (H&Y), and signed written informed consent before participation.

**Figure 1:**
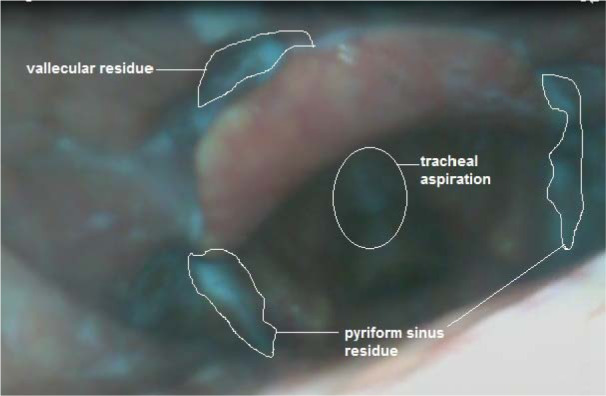
Baseline FEES showed a silent aspiration and moderate residues at both, the pyriform sinuses and vallecula for liquids.

**Figure 2:**
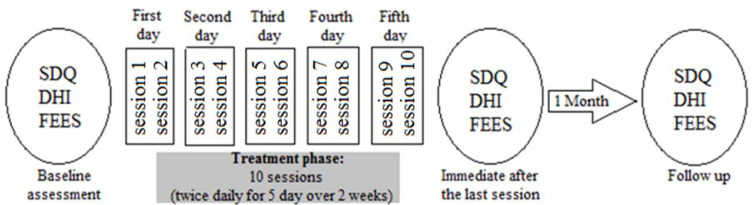
Treatment, and evaluation protocol. The patient received the combined treatment (Anodal tDCS, and conventional dysphagia treatment) for 5 days over two weeks, and was evaluated three times. Swallowing Disturbance Questionnaire (SDQ), Dysphagia Handicap Index (DHI) and Fiberoptic endoscopic evaluation of swallowing (FEES) were assessed at baseline, immediately after the last session, and one month after treatment.

Baseline swallowing status was assessed by Fiberoptic Endoscopic Evaluation of Swallowing (FEES), and the Swallowing Disturbance Questionnaire (SDQ) specifically developed for Parkinson's patients. Swallowing-related quality of life was assessed by the Dysphagia Handicap Index (DHI). FEES is performed using liquid, semi-solid, and solid textures with volumes of 5 and 10 ml, and swallowing is evaluated by the Penetration-Aspiration Scale (PAS) and scored between 1 (material does not enter the airway) and 8 (material enters the airway). The total score of the SDQ is between 0.5 and 44.5. A score of 12 or more is susceptive of dysphagia. The DHI questionnaire is a 25 item (score ranges between 0 (least disabled), and 100 (most disabled)) self-report questionnaire designed to measure the negative impact of dysphagia on quality of life. The scores of baseline assessments before the intervention were 17, and 32 for the SDQ and DHI scales, respectively. The PAS scores for liquids and solids were 8 and 2, respectively (Figure 1(Fig. 1)). The patient received anodal tDCS and Conventional Dysphagia Therapy (CDT) simultaneously for ten 30-minute sessions (twice daily for five days over two weeks) (Figure 2(Fig. 2)). In the first 20 minutes of each session, the patient received simultaneous tDCS and CDT, and in the last 10 minutes, CDT only was conducted. All sessions were conducted in the "on" phase of the PD medication cycle.

**Figure 3:**
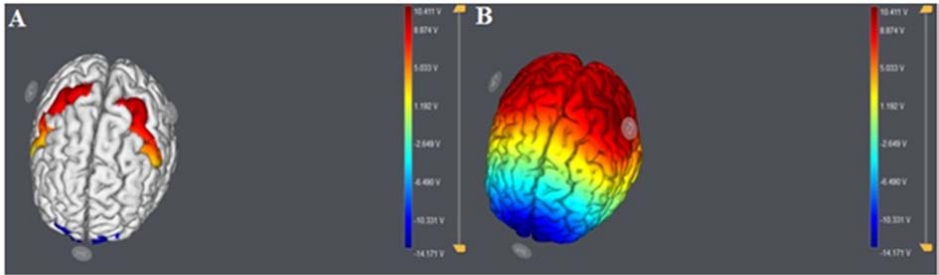
The Starstim system was used for modeling and stimulation. (A) Anodal (C3, C4) and return electrode (Fpz) placement and modeled electrical current flow (modeling conducted with the Neuroelectrics NAS software). (B) Bilateral anodal stimulation was applied over the PMC (C3 and C4 of the 10/20 system), and the return electrode was located at the supraorbital region (Fpz of the 10/20 system).

tDCS was delivered via the Starstim system (Neuroelectrics, Barcelona, Spain). Saline-soaked sponge-electrodes (35 cm^2^) were placed on the scalp. According to the 10-20 international EEG system, the two anodal electrodes were located over the pharyngeal motor cortex (PMC) (C3 and C4 positions of the left and right hemispheres). The return electrode was placed above the central supraorbital area (Fpz) (Figure 3(Fig. 3)). tDCS was applied twice daily with a 20 min interval in between for five days over two weeks. This spaced protocol has been shown to induce late phase plasticity effects before (Monte-Silva et al., 2013[6]). The stimulation intensity applied via each anodal electrode, and the duration of stimulation were 1 mA and 20 min, respectively. Both ramp-up and -down took about 10 seconds. The patient reported minor itching and tingling under the electrodes.

The outcome measures of this study were scores on the SDQ, PAS, and DHI. These assessments were performed three times, before the intervention, immediately after the last intervention session, and at the one-month follow-up. The results are summarized in Table 1(Tab. 1). The PAS, SDQ, and DHI scores decreased to 3 (for liquids), 4, and 12 after the last intervention, and were relatively stable (5 (for liquids), 5, and 16) at the one-month follow-up, respectively. The PAS score for solids did not change significantly after intervention.

## Discussion

These results provide the first evidence about the efficacy of anodal tDCS combined with conventional swallowing therapy as a novel therapeutic approach in PD. Our findings suggest that anodal tDCS over the PMC combined with conventional dysphagia treatment is feasible, safe, and well-tolerated, and has a significant clinical benefit compared to conventional therapy alone (Cosentino et al., 2018[2]).

The SDQ, DHI, and PAS scores obtained in the second and third assessments were relevantly lower compared to those in the first assessment, showing a clinically relevant effect of the intervention. For the third assessment, there was a slight trend for enhanced scores again, but the effects of the intervention were still present. These results are in line with those of Khedr et al., who investigated the effect of plasticity induction via rTMS on dysphagia in patients with Parkinson's disease (Khedr et al., 2019[3]). 

For mechanistic explanations of the observed effects, it can be assumed that motor cortex stimulation with the anode over the PMC results in an enhancement of the strength of swallowing-relevant synaptic connections in the cerebral cortex via increased activation of NMDA receptors in synergy with task-related receptor activation and a decrease in the inhibitory effect of GABA-ergic receptors (Stagg and Nitsche, 2011[9]; Michou and Hamdy, 2013[5]). Regarding parameters potentially relevant for the efficacy of the intervention, including the bilateral contribution of motor cortices to swallowing, the superior effects on online versus offline stimulation for motor learning and the superiority of spaced stimulation (Stagg et al., 2011[8]), we designed and tested an optimized combination of respective tDCS parameters for obtaining clinically relevant results. Given the mixed clinical effects of non-invasive brain stimulation for swallowing therapy so far, this protocol might be promising for achieving clinically relevant results in future clinical trials (Simons and Hamdy, 2017[7]). Hereby, an age-dependency of the effects of non-invasive brain stimulation on Parkinsonian patients, with better effects in younger patients, which has been recently reported, might have to be taken further into consideration for stimulation protocol adaptation in future studies (Málly et al., 2018[4]).

## Conclusion

In conclusion, the results of this study, although limited to a single case, suggest that spaced tDCS combined with CDT can improve both, swallowing functions and quality of life in PD patients. This study encourages further studies with randomized double-blinded design, more extended evaluations of the duration, and magnitude of effects, including the implementation of repeated intervention protocols suited to increase the stability of effects.

## Funding

This work was not supported by any funding from public, commercial or not-for-profit sectors.

## Conflict of interest

MAN is at the Scientific Advisory Boards of Neuroelectrics, and NeuroDevice. The other authors declare that they have no conflict of interest.

## Acknowledgement

Authors thank Tehran University of Medical Sciences for supporting this study. 

## Figures and Tables

**Table 1:**
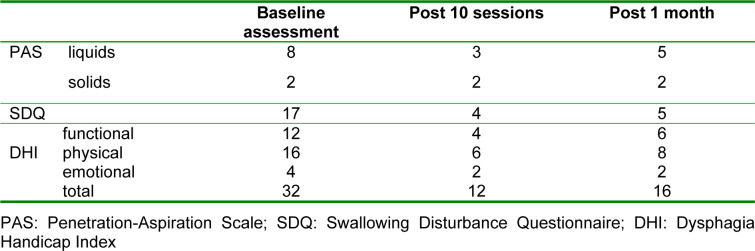
Summary of outcome measures
